# Predictors of response to methotrexate in juvenile idiopathic arthritis

**DOI:** 10.1186/1546-0096-12-35

**Published:** 2014-08-13

**Authors:** Mohamed Albarouni, Ingrid Becker, Gerd Horneff

**Affiliations:** 1 Centre of Paediatric Rheumatology, Department of General Paediatrics, Asklepios Klinik Sankt Augustin, Arnold-Janssen Str. 29, D-53757 Sankt Augustin, Germany; 2 Institute of Medical Statistics, Informatics and Epidemiology, University of Cologne, Cologne, Germany

## Abstract

**Background:**

The response to methotrexate so far is unpredictable in patients with juvenile idiopathic arthritis. Thus such predictors have to be determined in a large patient cohort.

**Methods:**

Demographic, clinical, articular and laboratory variables of patients newly treated with methotrexate were analysed by bivariate and logistic regression analysis to identify predictors of response to methotrexate. Minimal response was defined by the American College of Rheumatology pediatric (PedACR) 30 and strong response by the PedACR 70 criteria.

**Results:**

The patient population consisted of 731 patients. At month 3, 77.4% and at month 12 83.1% of patients were responders according to the PedACR 30 criteria, while 43.1% and 65.9% of patients had a PedACR 70 response at month 3 and at month 12. Thus minimal response was frequently already reached at month 3 while strong response to MTX treatment took usually longer to achieve.

In multivariate analysis the number of tender joints (p = 0.002), active joints (p < 0.001), concomitant use of NSAID (p = 0.027) and the parents evaluation of overall well-being (p < 0.001) were significant baseline parameters for minimal response at month 3, while at month 12 the determinants for reaching PedACR 70 were a disease duration < 1 year (p =0.001), a lower number of tender (p <0.001) but a higher number of active joints (p <0.001), a higher score of the parent’s evaluation of child’s pain (p =0.029), and the presence of morning stiffness (p =0.014).

**Conclusions:**

Baseline parameters for minimal response after 3 months of treatment and strong response after 12 months of treatment could be identified. Beside parameters defining activity and severity of disease, the disease duration and the concomitant use of NSAID were influencing factors. Overall the model of prediction could support physicians in making treatment decisions.

## Background

Juvenile idiopathic arthritis (JIA) is an umbrella term used to describe a heterogeneous group of disorders of unknown aetiology characterized by chronic arthritis affecting children below 16 years [1]. JIA is the most common chronic rheumatic illness in children and it is responsible for short and long-term disability [2]. In the recent years an increased number of disease-modifying anti-rheumatic drugs (DMARDs) have been developed for treatment of JIA, but methotrexate (MTX) is still the most common second line therapeutic agent used in treatment of JIA worldwide, either as monotherapy or in combination with biologic agents [3-10]. However, there is variation in the clinical response to MTX among the patients, while there does not appear to be any advantages related to efficacy or safety with either the oral or parenteral method of administration although it is believed by many pediatric rheumatologist that for severe JIA parenteral application may be an advantage [11]. Although serious toxicity in patients using MTX is uncommon, a prevalence of adverse effects as high as 42% has been reported [12,13]. The main goal of JIA treatment is the achievement of well being with minimal risk of side effects. Prediction of response can prevent further exposing of patients to side effects of MTX and also saving the time by progressing to the treatment with an alternative therapy (e.g. biological drugs) as soon as possible to prevent irreversible complications. Identification of predictors of response might also be helpful to develop recommendations for MTX use, especially starting of MTX as well as further continuation or early discontinuation and starting use of an alternative therapy [14-18]. Previous studies have shown conflicting results regarding predictors of response to MTX. In three previous studies, the results indicate a different effect of MTX according to the type of JIA. Halle and Prieur found that the systemic form seemed less responsive than ANA positive form with polyarticular course [19], while Woo et al. found that MTX is an effective treatment for both extended oligoarthritis and systemic JIA [20], Ravelli et al. concluded that the extended oligoarticular category is the best predictor of MTX efficacy [21]. However the analysis of the PRINTO MTX trial showed that the frequency of JIA categories was comparable between responders and non-responders [18]. The aim of this study is to determine whether demographic, clinical, articular and laboratory variables at baseline predict MTX response in patients with JIA.

## Methods

### Patient selection

Patient’s data were taken from the German BIKER Registry founded in 2001. The registry is a non-interventional long term study and has been approved by the ethics committee of the Aerztekammer Nordrhein, Duesseldorf, Germany. Since 2005 patients newly started with MTX were included in the registry. Inclusion criteria for our study were admittance to the registry until December 31, 2010, diagnosis of JIA according to the International League of Association for Rheumatology (ILAR) criteria [1], treatment with MTX just started, duration of MTX treatment of at least 3 months, and pretreatment data set available. Patients who received biologics were excluded.

### Evaluation of response to treatment

In this study early response to treatment was analysed at months 3 and additionally at 12 months. At each time the patients were divided into responders and non-responders according to the American College of Rheumatology Paediatric (PedACR) 30 or 70 improvement criteria [19], this means 30% or 70% improvement from baseline in at least three of any JIA core set variables (Physician’s and parent’s evaluation, number of active joints and joints with limited range of motion, Childhood Health Assessment Questionnaire [CHAQ] and erythrocyte sedimentation rate [ESR] ) with no more than one of the remaining variables worsened by more than 30%.

The predefined parameters with a potential influence on response included demographic (gender, age at onset of disease, age at start of treatment with MTX and disease duration until start of treatment), clinical (JIA category, global disease activity, pain assessment and child’s disability assessed by the Childhood Health Assessment Questionnaire disability index), articular (number of active joints, number of joints with limitation of motion [LOM], number of tender joints, presence of morning stiffness) and laboratory parameters (ESR, CRP, anti-nuclear antibody [ANA] and human leukocyte antigen [HLA] B27) [18-27].

### Statistics

Parameters are described as medians with first and third quartiles or mean ± standard deviation (SD) for quantitative variables and as absolute frequencies and percentages for qualitative variables. To detect relations between PedACR response and the potentially influencing parameters, Pearson or Spearman correlation coefficients were calculated, depending on parameter types. Multivariate logistic regression analyses were performed to evaluate the role of all factors that were significantly correlated with the response parameters (PedACR 30 or 70 responses at months 3 or 12). For the final model of each response parameter the adjusted odds ratios (OR) with confidence interval for the significant factors and the area under the receiver operating curve (AUC) were calculated. Analysis was performed using IBM® SPSS® Statistics version 21. The level of significance was set at 5%.

## Results

The screened total patient population in the registry consisted of 915 patients, 731 (79.9%) patients were treated for at least 3 months and had a full data set and therefore were eligible for the analysis at month 3. 707 could be identified for month 12 analyses. Females represent 68.7% of the cohort. Persistent oligoarthritis is the most common JIA category with 201 patients (28.3%) followed by RF negative polyarthritis with 200 patients (27.3%). Only 18 patients (2.5%) had unclassified arthritis. The mean age (+/− SD) at onset of the disease was 7.4+/−4.4 years (median 6.9 years; range 0.3-15.9 years). The mean age (+/−SD) at start of treatment in the total cohort was 9.5+/−4.7 years (median 9.9 years; range 1.1-18.1 years), while patients with systemic arthritis were youngest (mean +/− SD 6.99+/−4.05 years), patients with ERA-JIA were oldest (12.58+/−3.41 years) and others were in between (RF positive polyarthritis 12.58+/−3.41; RF negative polyarthritis 9.61 +/− 4.93; persistent oligoarthritis 8.02+/−4.27; extended oligoarthritis 9.01+/−4.22; psoriatic arthritis 10.5+/−4.69 and unclassified JIA 9.17+/−5.41).

The mean (+/−SD) of disease duration until start of treatment with MTX was 2.2+/−2.7 years (median 0.99 years; range 0.02-16.3 years). 91.1% patients have been treated with NSAID, and 21.2% patients had received oral corticosteroids while 34.6% patients have received intra-articular corticosteroids. The mean (+/−SD) of active joints at baseline was 5.9+/−7.5 (median 3), the mean of swollen joints was 4.9+/−6.8 (median 3), the mean number of tender joints 5.8+/−7.2 (median 3), while the mean of joints with limitation of movement was 5.7+/−7.5 (median 3). 59.2% of patients complained morning stiffness with a mean duration of 55.7+/−72.2 minutes (median 30 min).

At month 3, 77.4% of patients were responders according to the PedACR 30 criteria. This number increased to 83.1% at month 12. Thus, the majority of JIA patients of all JIA categories reached a minimal response according to the PedACR30 criteria already after 3 months of treatment. 43.1% and 65.9% of patients had a PedACR 70 response at month 3 and at month 12, respectively. The number of patients reaching a PedACR 70 at month 3 was markedly lower but dramatically increased after 12 months of treatment with MTX in all JIA categories, thus a 12 month duration of treatment was necessary to judge about a strong response (Table 1).

**Table 1:** Demographic, clinical, laboratory and articular characteristics at start of treatment in PedACR 30 responders and non- responders at month 3, PedACR 70 responders and non-responders at month 12

**Parameter**	**PedACR 30 at 3 months (n = 731)**		**PedACR 70 at 12 months (n = 707)**	
	**Responder**	**Non responder**	**Responder**	**Non responder**
	566 (77.4%)	165 (22.6%)	466 (65.9%)	241 (34.1%)
**Gender, female**	386 (68.2%)	116 (70.3%)	318 (68.2%)	172 (71.4%)
**Systemic onset JIA**	21 (84%)	4 (16%)	13 (76.5%)	4 (23.5%)
**RF-negative polyarthritis**	162 (81%)	38 (19%)	135 (70.3%)	57 (29.7%)
**RF-positive polyarthritis**	18 (81.8%)	4 (18.2%)	16 (69.6%)	7 (30.4%)
**Persistent oligoarthritis**	159 (76.8%)	48 (23.2%)	145 (67.4%)	70 (32.6%)
**Extended oligoarthritis**	71 (73.9%)	25 (26.1%)	63 (66.3%)	32 (33.7%)
**Enthesitis related arthritis**	66 (72.5%)	25 (27.5%)	52 (61.9%)	32 (38.1%)
**Psoriatic arthritis**	55 (76.4%)	17 (23.6%)	34 (50.7%)**	33 (49.3%)
**unclassified JIA**	14 (77.8%)	4 (22.2%)	8 (57.1%)	6 (42.9%)
**Age at onset of disease (years)**	6.8 (3.3-11)	6.4 (3.8-10.5)	6.1 (2.9-10.4)**	8.6 (3.9-12.1)
**Age at MTX start (years)**	9.7 (5.3-13.5)	9.5 (6–13.3)	8.5 (4.8-13)***	11.4 (6.8-14.5)
**Disease duration before MTX start (years)**	0.9 (0.3-2.9)	1.1 (0.4-3.2)	0.6 (0.3-2.3)***	1.3 (0.5-3.4)
**Concomitant use of NSAID**	514 (90.8%)**	149 (90.3%)	430 (92.3%)	221 (91.7%)
**Physician’s global assessment of disease activity**	40 (25–65)***	29 (19–51.7)	45 (26–67)***	30 (20–55)
**Parent’s global assessment of overall well-being**	44 (19–61)***	27 (8–46)	42 (20–59)**	31.5 (10–57)
**Parents evaluation of child’s pain**	40 (15–60.2)**	26.5 (4.2-53)	40 (17.2-58.7)**	29.5 (5–56.25)
**CHAQ-DI**	0.5 (0.12-0.87)***	0.25 (0–0.6)	0.5 (0.12-0.9)***	0.25 (0–0.75)
**HLA B27 positive**	94 (19.2%)	35 (22.9%)	77 (19.4%)	42 (18.8%)
**ANA positive**	271 (49.2%)	76 (48.4%)	240 (53.7%)	117 (48.9%)
**ESR (mm/h)**	18 (10.0-31)***	12 (6.0-23.5)	19.5 (10–36)***	12 (6.5-24)
**CRP (mg/dl)**	4.2 (1.1-12)	3 (1–7)	5 (2–15.35)***	3 (1–7)
**No. of active joints**	4 (2–8)***	2 (1–3)	4 (2–8)***	2 (1–5)
**No of tender joints**	3 (2–7)***	2 (1–4 )	3 (2–7)**	2 (1–6)
**No. of swollen joints**	4 (2–8)***	2 (1–4)	3 (2–7)***	2 (0–5)
**No. of joints with LOM**	4 (2–8)***	2 (1–4)	4 (2–8)***	2 (1–5)
**Presence of morning stiffness**	360 (63.6%)***	71 (43%)	300 (64.4%)***	120 (49.8%)

Data are numbers (%) or median (first to third quartile). P-values of correlation with PedACR 30 or 70 are shown in column “responder”.

**p < 0.01;***p < 0.001.

After 3 months, response according to the PedACR30 criteria was reached by 72.5% of JIA patients with enthesitis related arthritis up to 84% in JIA patients with systemic arthritis. No significant difference was noted here between the JIA categories. After 12 months of treatment, response according to the PedACR70 criteria was reached by 50.7% of JIA patients with psoriatic arthritis up to 76.5% in JIA patients with systemic arthritis. In pairwise analysis only, but not in overall ANOVA, significant lower numbers of patients with psoriatic arthritis JIA reached high level of response. No further significant difference was noted here between the JIA categories.

A full data set at month 3 and month 12 was available for 562 (61.4%) patients. 319 patients (56.4%) of the total PedACR 30-responders (total number n = 566) at month 3 were PedACR 70-responders at month 12. 123 (21.7%) of the PedACR30 responders at month 3 failed to reach a PedACR70 at month 12. 124 (21.9%) patients did yet not reach the 12 month point of time or had an incomplete data set. 73 patients (44.2% ) of those who failed a PedACR 30-response (total number n = 165) at month 3 also failed to reach a PedACR70 at month 12 while only 47 (28.5%) reached a PedACR70 at month 12. The remaining 45 (27.3%) patients had missing data.

Thus reaching a minimal response at month 3, demonstrated by achievement of the PedACR30 is highly predictive for achieving a strong response at month 12 demonstrated by the PedACr70 criteria (Odd’s ratio 4.03 [95% CI 2.64-6.14]; p < 0.001, χ2-test).

### Bivariate analysis

#### Minimal response (PedACR 30)

The concomitant use of NSAID, presence of morning stiffness, higher numbers of active, tender and swollen joints as well as numbers of joints with LOM, higher disability score in the CHAQ, higher parent’s global assessment of overall well-being, physician’s global assessment of disease activity and ESR value at baseline were associated with minimal response (PedACR 30) at months 3 and also at 12, while a higher score of parent’s evaluation of child’s pain (VAS-Global) was predictive for an ACR30 response at month 3, and a shorter disease duration before MTX start, a lower age at onset of disease and at MTX start were also associated with minimal response (PedACR 30) at month 12.

The median disease duration before start of treatment in PedACR 30 responders was 0.9 (0.3-2.9) years at month 3 and 0.7 (0.3-2.7) years at month 12. Non-responders at month 3 or 12 had a significantly higher disease duration compared to responders. At month 3, 54.5% of non-responders and at month 12, 63% of non-responders had a disease duration of more than 1 year (Table 1).

#### Strong response (PedACR 70)

A shorter disease duration before MTX start, higher numbers of active, swollen, tender joints as well as numbers of joints with LOM, a higher disability score in the CHAQ, higher Parent’s global assessment of overall well being, physician’s global assessment of disease activity and ESR value at baseline were associated with strong responses (PedACR 70) at months 3 and 12. A lower age at onset of disease and at MTX start, a higher score of parent’s evaluation of child’s pain (VAS-Global), a higher CRP value and the presence of morning stiffness were associated with strong response (PedACR 70) at month 12 only. The rheumatoid factor positive polyarthritis category was a positive predictor at month 3, while psoriasis associated arthritis was a negative predictor at month 12.

The median disease duration before start of treatment in PedACR 70 responders was 0.8 (0.3-2.7) years at month 3 and 0.6 (0.3-2.3) at month 12. Non-responders at month 3 or 12 had a significantly higher median disease duration of 1 and 1.3 years, respectively, compared to responders. At month 3, 53% of non-responders and at month 12, 65.9% of non-responders had a disease duration of more than 1 year.

### Multivariate analysis

**Figure 1:**
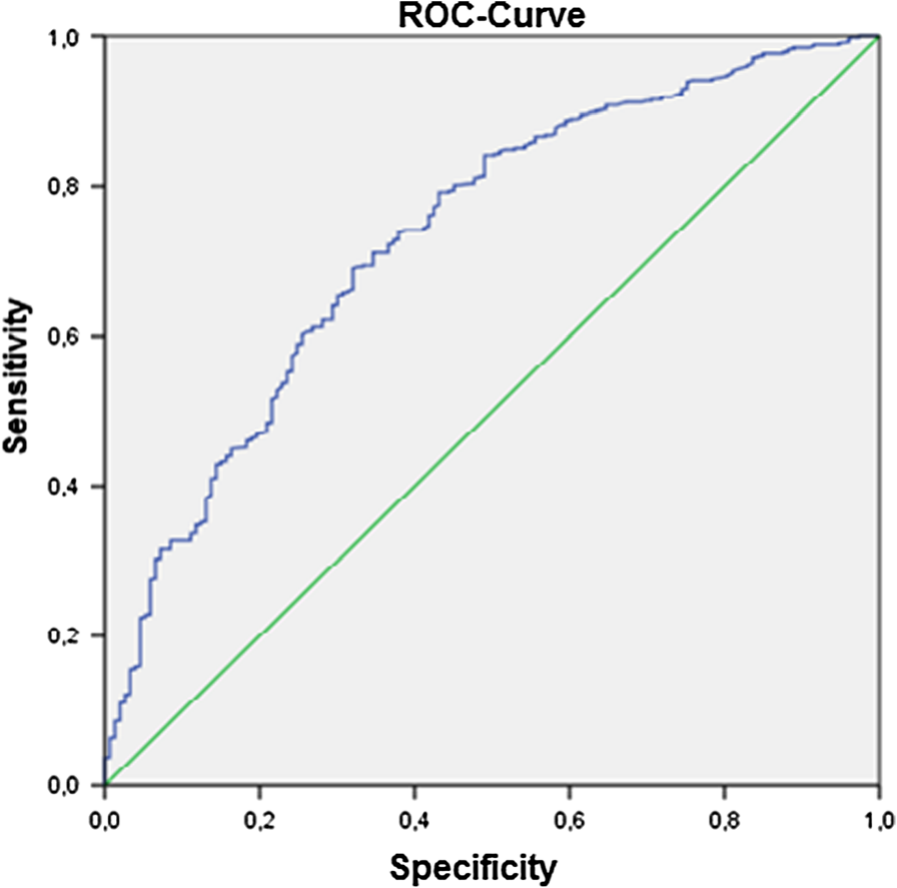
ROC-Curve for PedACR 30 at month 3 (AUC = 0,736, specificity = 12.4%, sensitivity = 98.7%).

**Figure 2:**
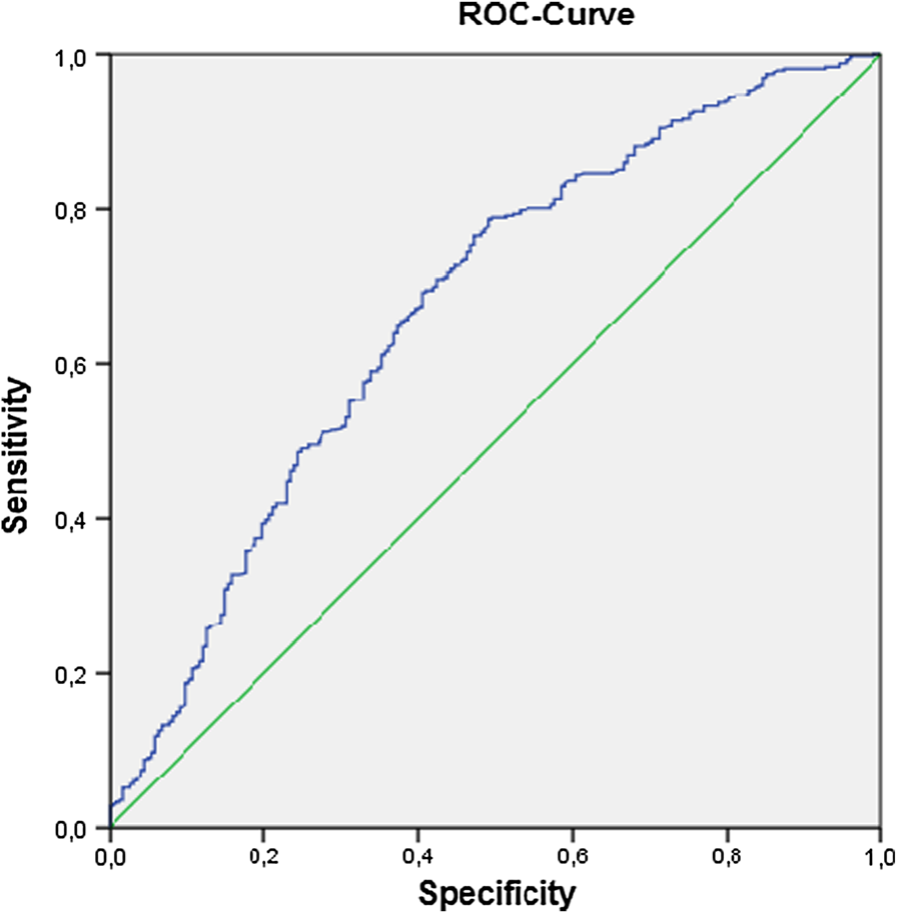
ROC-Curve for PedACR 70 at month 12 (AUC = 0,672, specificity = 27%, sensitivity = 90.7%).

Multivariate logistic regression analysis was performed with all variables that did significantly correlate with PedACR 30 at month 3 and PedACR 70 response at month 12. The predictor’s accuracy was evaluated by ROC (receiver operating characteristic) curve analysis. The results for PedACR 30 at month 3 yielded an AUC of 0.736 with a specificity of 12.4% and a sensitivity of 98.7% (Table 2). For the PedACR 70, we found a reasonable regression model at month 12 (AUC = 0.672, specificity =27.0% and sensitivity = 90.7%). The model at month 3 showed accuracy ≤60%, which is barely above chance, and therefore not useful as prediction model (Figures 1 and 2).

**Table 2:** Final logistic regression models for PedACR 30 at month 3 and PedACR70 at month 12 with adjusted odds ratios

**Determinants of response**	**OR (95% CI)**	**p-value**
***PedACR 30 at month 3***		
No. of tender joints*	0.92 (0.88-0.97)	0.002
No. of active joints*	1.26 (1.16-1.36)	<0.001
Parents global assessment of overal well-being [VAS 0-100 mm]*	1.02 (1.01-1.03)	<0.001
Concomitant use of NSAIDs	1.89 (1.08-3.34)	0.027
Model performance:		
AUC	73.6%	
Sensitivity	98.7%	
Specificity	12.4%	
***Ped ACR70 at month 12***		
Disease duration >1 year	0.54 (0.39-0.77)	0.001
No. of tender joints*	0.92 (0.88-0.97)	<0.001
No. of active joints*	1.10 (1.05-1.16)	<0.001
Parent’s global assessment of child’s pain [VAS 0-100 mm]*	1.01 (1.00-1.01)	0.029
Presence of morning stiffness	1.58 (1.10-2.28)	0.014
Model performance:		
AUC	67.2%	
Sensitivity	90.7%	
Specificity	27.0%	

Specificity = probability for non-responders being correctly identified by the model, sensitivity = likewise defined probability for responders. AUC = area under the curve, OR = odd’s ratio, *per increment of 1 unit.

## Discussion

The aim of modern treatment for JIA is the rapid induction of disease control to prevent joint damage, maximize physical function and to promote normal growth and normal lifestyle for the patients, as well as to achieve these goals with minimal risk of side effects. The recent concept of JIA treatment suggests that the early aggressive intervention may buy long term disease suppression [18]. The aim of this retrospective study on a high number of patients with JIA, who were treated with MTX for at least 3 months, was to determine the predictors of response to MTX. Despite the presence of considerable variation in the clinical response to MTX among patients, and the availability of several new agents for the treatment of JIA, MTX remains the most common second line therapeutic agent used in treatment of JIA worldwide because of both cost and experience [3-7]. The identification of predictors of response is helpful to develop recommendations for MTX use, especially starting of MTX as well as further continuation or early discontinuation and starting use of biological drugs. Thus, the aim of this study is to determine whether demographic, clinical, articular and laboratory variables at baseline can predict MTX response in patients with JIA.

The PedACR 30 was initially designed to distinguish between active treatment and placebo, it was a significant step towards creating standardized outcome measures in paediatric rheumatology, primarily assessing relative efficacy within the context of clinical trials. These measures are of less utility in quantifying response, tracking patient progress longitudinally and describing an individual’s disease state at a specific moment [28]. In this study the achievement of PedACR 30 at month 3 was a highly significant positive predictor for reaching PedACR 70 at month 12 with an odd’s ratio of 4.03 [95% CI 2.64-6.14].

It has been argued that only improvement in disease activity above PedACR 70 predicts a favourable long term outcome and reflects a major clinical response to treatment [18,29].

The present study confirms the observations of the PRINTO study regarding JIA categories[ [18]. In the multivariate analyses JIA categories did not significantly predict the response to MTX according to PedACR 70 criteria.

The PedACR 70-response rate in our study increased markedly from month 3 to month 12. The increasing number of responders in the course of treatment suggests that the low success at three months may be affected by the delay of clinical response achieved by MTX treatment. These findings are in line with common view that the maximum therapeutic effect usually becomes apparent 4 to 6 months after the beginning of treatment [5].

After 12 months from the beginning of therapy with MTX, the strong response according to PedACR 70 was associated with disease duration less than 1 year, higher numbers of active joints, lower numbers of tender joints, higher scores for parent’s evaluation of pain and the presence of morning stiffness. The presence of the number of active joints as a part of the PedACR score of improvement could hardly explain the relationship, which most likely is due to anti-inflammatory mechanism of action of MTX. This may explain the relationship between reaching of strong response and the number of active joints as well as other activity parameters such as morning stiffness and pain, which in the present study was assessed by patient or parents global assessment of pain. In contrast, higher numbers of tender joints were predictive for poor response to MTX at month 12. The significant relationship between the number of tender joints and reaching a PedACR 70 response can be explained by the indirect effect of presence of tenderness or pain on the components of PedACR criteria, not only on physician’s global assessment score and parent’s global assessment of overall well-being, but also on CHAQ-DI, which comprises two indices, the first is the disability index, and the other is discomfort index which is determined by the presence of pain measured by a 100 mm analogue scale [30].

Disease duration before MTX start was significantly associated with PedACR 70 response in bivariate analysis, but in general the disease duration as a variable was not significant in multivariate analysis. This may be because disease duration was highly correlated with the age at start of MTX therapy. After repeating multivariate analysis with exclusion of age at MTX start, the categorized disease duration (less or more than 1 year) had a significant effect on reaching PedACR 30 and 70. We found that a shorter disease duration (<1 year) was significantly associated with reaching a strong response (PedACR 70) at month 12, while higher disease duration (>1 year) was predicted a poor response. This finding supports the results of the PRINTO study and the previous clinical experience which suggests that the early treatment is more effective [18,27,31].

In contrast to the PRINTO study of predictors of response to MTX, in our study the presence of ANA as well as CHAQ score were no significant predictors for a strong response (PedACR 70) [18]. These variations may be due to many differences between the present study and the PRINTO study, although the general designs were similar. Bivariate and logistic regression analysis was used to identify baseline predictors of poor response. Also the improvement was assessed according to the American College of Rheumatology criteria for pediatrics, by using PedACR 30 for minimal improvement analysis and PedACR 70 for strong improvement analysis. In the PRINTO study however, the patients with seropositive polyarthritis, psoriatic arthritis and enthesitis related arthritis categories were excluded from the study sample, while in the present study all JIA categories were included. An important difference between the two studies is the time of evaluation of improvement, while in the PRINTO study the evaluation of improvement was done at month 6 only, in our study the evaluation was done at month 3 and 12. This gives advantages to the present study and allows for better assessment of early response as well as the response after long duration of treatment and determination which variables could predict the response at these times. Another advantage of the present study is the larger study. Our sample included 731 patients, while in the PRINTO study the sample was restricted to 563 patients.

The diagnostic accuracy was not very well. The AUC in the ROC analysis for PedACR 30 at month 3 was 0.73, while for PedACR 70 at month 12, the AUC was 0.67. The results can be accepted especially when compared with results of other studies. These values are slightly better than the results of the PRINTO study, in which AUC for PedACR 30 was 0.65, and for PedACR 70 was 0.66 [18], possibly because of the higher patient number.

## Conclusion

We have found that a longer disease duration, a lower number of active joints, a higher number of tender joints, a lower score of parent’s evaluation of child’s pain at baseline were significantly at risk not to achieve a PedACR 70 response, while the presence of morning stiffness was a positive predictor to reach PedACR 70. Interstingly, early PedACR 30 responders were much more likely to reach a strong response later on. These findings can be considered as recommendations for the use of MTX in patients with JIA, since the presence of these baseline determinants predict a response to MTX, even after prolongation of exposure for up to 12 months, thereby may prompt the physicians to start an alternative drug therapy earlier.

### Consent

Written informed consent was obtained from the parents and/or patients for the survey, evaluation and publication of this report.

## Competing interests

The authors declare that they have no competing interests.

## Authors’ contributions

MA drafted the manuscript. GH designed and carried out the study and drafted the manuscript. IB carried out the statistical analyses and drafted the manuscript. All authors read and approved the final manuscript.
